# GLP‐1 Receptor Agonists and Risk of Skin Cancer in Adults With Type 2 Diabetes: A Target Trial Emulation

**DOI:** 10.1111/dom.71142

**Published:** 2026-07-28

**Authors:** Huilin Tang, Bingyu Zhang, Ying Lu, Ting Zhou, Yiwen Lu, César A. Briceño, Jiali Han, Yong Chen

**Affiliations:** ^1^ The Center for Health AI and Synthesis of Evidence (CHASE) University of Pennsylvania Philadelphia Pennsylvania USA; ^2^ Department of Biostatistics, Epidemiology, and Informatics University of Pennsylvania Perelman School of Medicine Philadelphia Pennsylvania USA; ^3^ Center for Preventative Ophthalmology and Biostatistics, Department of Ophthalmology University of Pennsylvania Perelman School of Medicine Philadelphia Pennsylvania USA; ^4^ The Graduate Group in Applied Mathematics and Computational Science, School of Arts and Sciences University of Pennsylvania Philadelphia Pennsylvania USA; ^5^ Department of Ophthalmology, Scheie Eye Institute University of Pennsylvania Perelman School of Medicine Philadelphia Pennsylvania USA; ^6^ Department of Epidemiology, Richard M. Fairbanks School of Public Health Indiana University Indianapolis Indiana USA; ^7^ Leonard Davis Institute of Health Economics University of Pennsylvania Philadelphia Pennsylvania USA; ^8^ Penn Medicine Center for Evidence‐Based Practice (CEP) University of Pennsylvania Philadelphia Pennsylvania USA; ^9^ Penn Institute for Biomedical Informatics (IBI) University of Pennsylvania Philadelphia Pennsylvania USA

**Keywords:** cohort study, GLP‐1 analogue, incretin therapy, pharmaco‐epidemiology, type 2 diabetes

## Abstract

**Aims:**

Existing evidence regarding the association between glucagon‐like peptide‐1 receptor agonists (GLP‐1RAs) and risk of melanoma and non‐melanoma skin cancer (NMSC) is inconclusive. This study aimed to assess the association between GLP‐1RA initiation and risks of melanoma and NMSC versus sodium–glucose cotransporter 2 inhibitors (SGLT2is) and dipeptidyl peptidase 4 inhibitors (DPP4is) in adults with type 2 diabetes (T2D).

**Materials and Methods:**

Target trial emulation using TriNetX electronic health records (2014–2025). Adults with T2D initiating GLP‐1RAs, SGLT2is or DPP4is were included. Outcomes were incident melanoma and NMSC over 3 years. Propensity score matching and Cox models estimated hazard ratios (HRs). Sensitivity analyses included negative control outcome (NCO) calibration, a lag‐period analysis and restriction to patients with obesity.

**Results:**

After matching, the GLP‐1RA versus SGLT2i cohort included 235 797 pairs; the GLP‐1RA versus DPP4i cohort included 153 873 pairs. Mean follow‐up ranged from 1.8 to 2.2 years. GLP‐1RA use was not associated with melanoma compared with either SGLT2i (HR, 1.04; 95% CI, 0.93–1.17) or DPP4i (HR, 1.09; 95% CI, 0.95–1.25). A borderline increase in NMSC risk was observed in the comparison with SGLT2is (HR, 1.06; 95% CI, 1.00–1.11); no such association was identified in the comparison with DPP4is (HR, 1.03; 95% CI, 0.97–1.09). Findings were generally consistent across sensitivity analyses; however, the association with NMSC was attenuated after NCO calibration.

**Conclusions:**

GLP‐1RA therapy was not associated with an increased risk of melanoma. A borderline increase in NMSC risk was observed compared with SGLT2is but was not evident after NCO calibration.

## 1 Introduction

Glucagon‐like peptide‐1 receptor agonists (GLP‐1RAs) are used widely for the management of type 2 diabetes (T2D) and, increasingly, obesity [1, 2]. Several GLP‐1RAs have demonstrated cardiovascular and renal protective effects, and their role in metabolic disease treatment continues to expand [3]. As prescribing volumes increase, evaluating their long‐term safety profile is essential.

Concerns have been raised regarding potential dermatologic adverse events, specifically melanoma and non‐melanoma skin cancer (NMSC) [4, 5]. GLP‐1 receptors are expressed in multiple tissues, including human melanocytes, raising the possibility of direct biological effects [6]. Preclinical studies have reported the activation of potentially mitogenic signalling pathways in melanocytes and keratinocytes following GLP‐1RA exposure [7, 8, 9], leading to uncertainty about their carcinogenic potential [6, 10, 11]. In addition, an increased risk of skin cancer was observed in a large cardiovascular outcome trial of liraglutide, a commonly used GLP‐1RA [12]. Several observational studies and pharmacovigilance analyses have reported possible associations between GLP‐1RA use and skin cancer risk [4, 5]; however, findings have been inconsistent and frequently limited by residual confounding.

To address these gaps, we conducted a large, multi‐institutional, real‐world study using a target trial emulation framework and robust both measured and unmeasured confounding adjustment, including propensity score matching (PSM) and negative control outcome (NCO) calibration. We examined whether initiation of GLP‐1RAs is associated with an increased incidence of melanoma or NMSC compared with two commonly prescribed alternative glucose‐lowering drugs: sodium–glucose cotransporter 2 inhibitors (SGLT2i) and dipeptidyl peptidase 4 inhibitors (DPP4i).

## 2 Methods

### 2.1 Study Design and Data Source

We conducted a target trial emulation using deidentified electronic health record (EHR) data from the TriNetX Research Network, a global collaborative network of academic and community health systems that aggregates standardised, longitudinal clinical data, including demographics, diagnoses, procedures, laboratory values, vitals, medications and mortality data [13]. For this study, patient‐level data from participating healthcare organisations across the United States were extracted and analysed locally (from database inception through January 31, 2025) to ensure methodological transparency and reproducibility, rather than relying solely on platform‐based aggregate queries. Because all data were deidentified, the study was approved by University of Pennsylvania Institutional Review Board (IRB # 858496) and did not require informed consent. Key elements of the emulated target trial protocol are summarised in Table S1. This study was reported following the Strengthening the Reporting of Observational Studies in Epidemiology (STROBE) reporting guideline [14].

### 2.2 Stuty Population

Among adults aged 18 years or older with at least one diagnosis code for T2D, identified using the International Classification of Diseases, 10th Revision, Clinical Modification (ICD‐10‐CM; E11), we identified new users of GLP‐1RAs, SGLT2i or DPP4i between 1 January 2014, and 31 January 2025, occurring on or after each patient's initial diagnosis of T2D. New use was defined as a first prescription for the respective drug class with no prescriptions for that class in the preceding 12 months. The index date was defined as the date of drug initiation. Patients were assigned exclusively to the first GLP‐1RA, SGLT2i or DPP4i they initiated. Two active‐comparator new‐user cohorts were constructed: GLP‐1RA versus SGLT2i initiators and GLP‐1RA versus DPP4i initiators. For each comparison, individuals initiating agents from both classes on the index date were excluded. Patients with any prior diagnosis of melanoma or NMSC were excluded. Additional exclusions included type 1 diabetes and end‐stage kidney disease (ESKD) or dialysis.

### 2.3 Exposure, Outcomes and Follow‐Up

Exposures of interest were GLP‐1RAs, including liraglutide, semaglutide, dulaglutide, exenatide, lixisenatide and tirzepatide in any formulation, compared with any SGLT2i or DPP4i agent (Table S2). Patients were categorised according to the first agent they initiated and were analysed following an intention‐to‐treat (ITT) approach, whereby exposure status was fixed at baseline regardless of subsequent treatment changes or discontinuation.

The primary outcomes were incident melanoma (ICD‐10‐CM: C43) and incident NMSC (ICD‐10‐CM: C44), defined as using ≥ 1 ICD diagnosis codes with a positive predictive value of 58% and 98%, respectively (Table S3) [15]. An iITT framework was applied to preserve baseline comparability between exposure groups, minimise bias from post‐index treatment modifications and reflect real‐world treatment effectiveness. Follow‐up began on the index date and continued until the earliest of first melanoma or NMSC diagnosis, death, loss to follow‐up (last encounter date), 3 years after the index date or end of study (31 January 2025). Melanoma and NMSC were analysed as separate outcomes. A maximum follow‐up of 3 years was selected to balance sufficient time for potential skin cancer development with the need to limit exposure misclassification, informative censoring and secular changes in treatment patterns over longer follow‐up periods.

### 2.4 Covariates

Baseline covariates were assessed during the 12 months preceding the index date and are summarised in Table 1. Demographic characteristics included age, sex, race and ethnicity, geographic region and calendar year of cohort entry. We also evaluated a comprehensive set of comorbidities relevant to both diabetes severity and skin cancer risk. These included diabetic microvascular and macrovascular complications, cardiovascular disease, hypertension, hyperlipidemia, chronic kidney disease and psychiatric disorders. Social determinants of health were incorporated based on diagnostic codes reflecting socioeconomic risk factors and lifestyle‐related challenges (e.g., housing or financial insecurity, and problems related to physical activity or diet). Lab and vital values, such as body mass index (BMI), haemoglobin A1c (HbA1c), systolic and diastolic blood pressure, estimated glomerular filtration rate (eGFR) and lipid profiles, were included to characterise baseline metabolic and cardiovascular risk. Missing data for lab and vital values were imputed using multiple imputation by chained equations, including all baseline covariates [16].

**TABLE 1:** The risk of incident melanoma and non‐melanoma skin cancer (up to 3‐year follow‐up) in patients initiating GLP‐1RA versus SGLT2i and GLP‐1RA versus DPP4i.

Outcomes	GLP‐1RA vs. SGLT2i		GLP‐1RA vs. DPP4i	
	GLP‐1RA	SGLT2i	GLP‐1RA	DPP4i
Melanoma				
Events/Patients at risk, *n*/*N*	583/235797	557/235797	431/153873	389/153873
Mean follow‐up (SD), years	1.82 (1.07)	1.82 (1.09)	2.23 (1.01)	2.20 (1.05)
IR, per 1000 person‐years	1.36 (1.25, 1.47)	1.30 (1.20, 1.41)	1.25 (1.14, 1.38)	1.15 (1.04, 1.27)
RD, per 1000 person‐years	0.06 (−0.10, 0.22)	Reference	0.11 (−0.06, 0.27)	Reference
1:1 PSM HR (95% CI)	1.04 (0.93, 1.17)	Reference	1.09 (0.95, 1.25)	Reference
Apply 1‐year lag period	1.09 (0.91, 1.31)	Reference	0.99 (0.81, 1.20)	Reference
NCO calibrated HR (95% CI)	0.97 (0.78, 1.21)	Reference	1.06 (0.86, 1.30)	Reference
Non‐melanoma skin cancer				
Events/patients at risk, *n*/*N*	3034/235797	2868/235797	2289/153873	2186/153873
Mean follow‐up (SD), years	1.81 (1.07)	1.80 (1.09)	2.22 (1.02)	2.19 (1.05)
IR, per 1000 person‐years	7.13 (6.88, 7.39)	6.74 (6.50, 6.99)	6.71 (6.44, 6.99)	6.50 (6.23, 6.78)
RD, per 1000 person‐years	0.38 (0.03, 0.74)	Reference	0.21 (−0.18, 0.60)	Reference
1:1 PSM HR (95% CI)	1.06 (1.00, 1.11)	Reference	1.03 (0.97, 1.09)	Reference
Apply 1‐year lag period	1.10 (1.02, 1.18)	Reference	1.07 (0.98, 1.15)	Reference
NCO calibrated HR (95% CI)	0.98 (0.82, 1.19)	Reference	1.00 (0.84, 1.18)	Reference

Abbreviations: CI, confidence interval; DPP4i, dipeptidyl peptidase‐4 inhibitor; GLP‐1RA, glucagon‐like peptide‐1 receptor agonist; HR, hazard ratio; IR, incidence rate; NCO, negative control outcome; PSM, propensity score matching; SGLT2i, sodium–glucose cotransporter‐2 inhibitors.

### 2.5 Statistical Analysis

For each comparator cohort, propensity scores were estimated using logistic regression models with treatment group as the dependent variable and all baseline covariates as predictors. We applied 1:1 nearest‐neighbour PSM without replacement, using a calliper width of 0.1 of the standard deviation of the logit of the propensity score. Covariate balance between treatment groups was assessed using standardised mean differences (SMDs), with an SMD < 0.10 indicating adequate balance across matched cohorts [17]. In the matched cohorts, we calculated incidence rate (IR) and rate difference (RD) for melanoma and NMSC. Additionally, Cox proportional hazards models were applied to estimate hazard ratios (HRs) and 95% confidence intervals (CIs) for the association between GLP‐1RA initiation and each outcome, incorporating robust sandwich variance estimators to account for the matched design [18]. Kaplan–Meier curves were generated to depict cumulative incidence over the follow‐up period.

Several sensitivity analyses were conducted to evaluate the robustness of the findings: First, to assess and adjust for residual confounding, a set of NCOs not plausibly affected by GLP‐1RA exposure (e.g., abnormal pupil and tooth loss) (Table S4) was analysed using the same design and analytic approach as the primary analysis. Effect estimates from NCOs were used to construct an empirical null distribution, which was subsequently applied to calibrate HRs and 95% CIs for the primary outcomes using established empirical calibration methods [19, 20, 21]. Second, to enhance biological plausibility and reduce potential bias from reverse causation and pre‐existing but undiagnosed cancers, a lag‐period analysis was performed. Specifically, a 1‐year lag period was applied, whereby outcomes occurring within the first year after treatment initiation were excluded, and follow‐up for outcomes began 365 days after cohort entry. Prespecified subgroup analyses were conducted to evaluate potential heterogeneity in treatment effects across clinically relevant patient characteristics, including age group, sex, race and ethnicity, obesity status, baseline insulin use and individual GLP‐1RA agent. Additional analyses were performed among patients with obesity. All analyses were performed using R, version 4.5.0 (R Foundation for Statistical Computing), and statistical significance was defined as a two‐sided *p* < 0.05.

## 3 Results

According to the inclusion and exclusion criteria, we identified 407 709 adults with T2D who initiated a GLP‐1RA and 316 488 who initiated an SGLT2i in the GLP‐1RA versus SGLT2i cohort, and 408 393 GLP‐1RA initiators and 239 701 DPP4i initiators in the GLP‐1RA versus DPP4i cohort (Figure S1). Before matching, GLP‐1RA initiators differed substantially from both SGLT2i and DPP4i initiators, being younger, more often women, more likely to have obesity and sleep or mood disorders (Tables S5 and S6). After performing 1:1 PSM within each comparison, all baseline characteristics were well balanced (all SMDs < 0.10), yielding 235 797 matched pairs in the GLP‐1RA versus SGLT2i cohort and 153 873 matched pairs in the GLP‐1RA versus DPP4i cohort (Tables S5 and S6). The distribution of propensity score before and after PSM is shown in Figure S2. Across the matched cohorts, the mean age ranged from 60.61 to 61.41 years, 44.4% to 50.9% were women and the mean BMI ranged from 33.01 to 33.32 kg/m^2^. Mean follow‐up ranged from 1.8 to 2.2 years.

### 3.1 Primary Results

**FIGURE 1:**
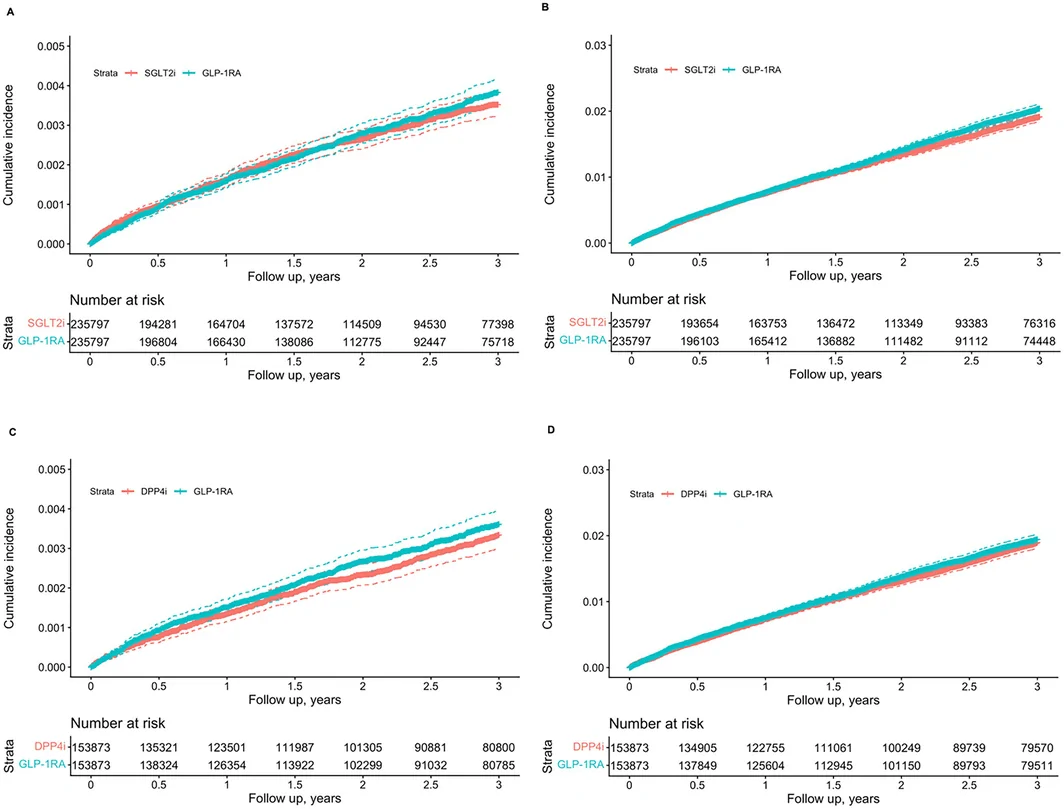
Propensity score matched cumulative incidence for melanoma and non‐melanoma skin cancer in patients with type 2 diabetes initiating GLP‐1RA versus SGLT2i and GLP‐1RA versus DPP4i. (A) Melanoma (GLP‐1RA vs. SGLT2i). (B) Non‐melanoma skin cancer (GLP‐1RA vs. SGLT2i). (C) Melanoma (GLP‐1RA vs. DPP4i). (D) Non‐melanoma skin cancer (GLP‐1RA vs. DPP4i). DPP4i, dipeptidyl peptidase‐4 inhibitor; GLP‐1RA, glucagon‐like peptide‐1 receptor agonist; SGLT2i, sodium–glucose cotransporter‐2 inhibitors.

During the follow‐up, 583 of 235 797 GLP‐1RA initiators and 557 of 235 797 SGLT2i initiators developed melanoma, yielding an IR of 1.36 (95% CI, 1.25–1.47) and 1.30 (95% CI, 1.20–1.41) per 1000 person‐years, respectively. The RD was 0.06, per 1000 person‐year (95% CI, −0.10 to 0.22) (Table 1). The Kaplan–Meier curves are presented in Figure 1. GLP‐1RA initiation was not associated with an increased risk of melanoma compared with SGLT2i (HR, 1.04; 95% CI, 0.93–1.17). Similarly, compared with DPP4i initiation, GLP‐1RA initiation was not associated with an increased risk of melanoma, with an RD of 0.11 (95% CI, −0.06 to 0.27) per 1000 person‐years, and an HR of 1.09 (95% CI, 0.95–1.25).

For NMSC, 3034 of 235 797 GLP‐1RA initiators and 2868 of 235 797 SGLT2i initiators developed NMSC during the follow‐up, corresponding to an RD of 0.38 per 1000 person‐years (95% CI, 0.03–0.74) (Table 1). GLP‐1RA initiation was associated with a borderline increase in the risk of NMSC compared with SGLT2i (HR, 1.06; 95% CI, 1.00–1.11) (Figure 1). However, when compared with DPP4i initiation, GLP‐1RA initiation was not associated with an increased risk of NMSC, with an RD of 0.21 (95% CI, −0.18 to 0.60) per 1000 person‐years and an HR of 1.03 (0.97 to 1.09).

### 3.2 Sensitivity and Subgroup Analyses

The results were consistent after applying 1‐year lag period (Table 1). After applying NCO calibration, the HRs were attenuated, with no significant difference in NMSC risk between GLP‐1RAs and SGLT2is (HR, 0.98; 95% CI, 0.82–1.19).

**FIGURE 2:**
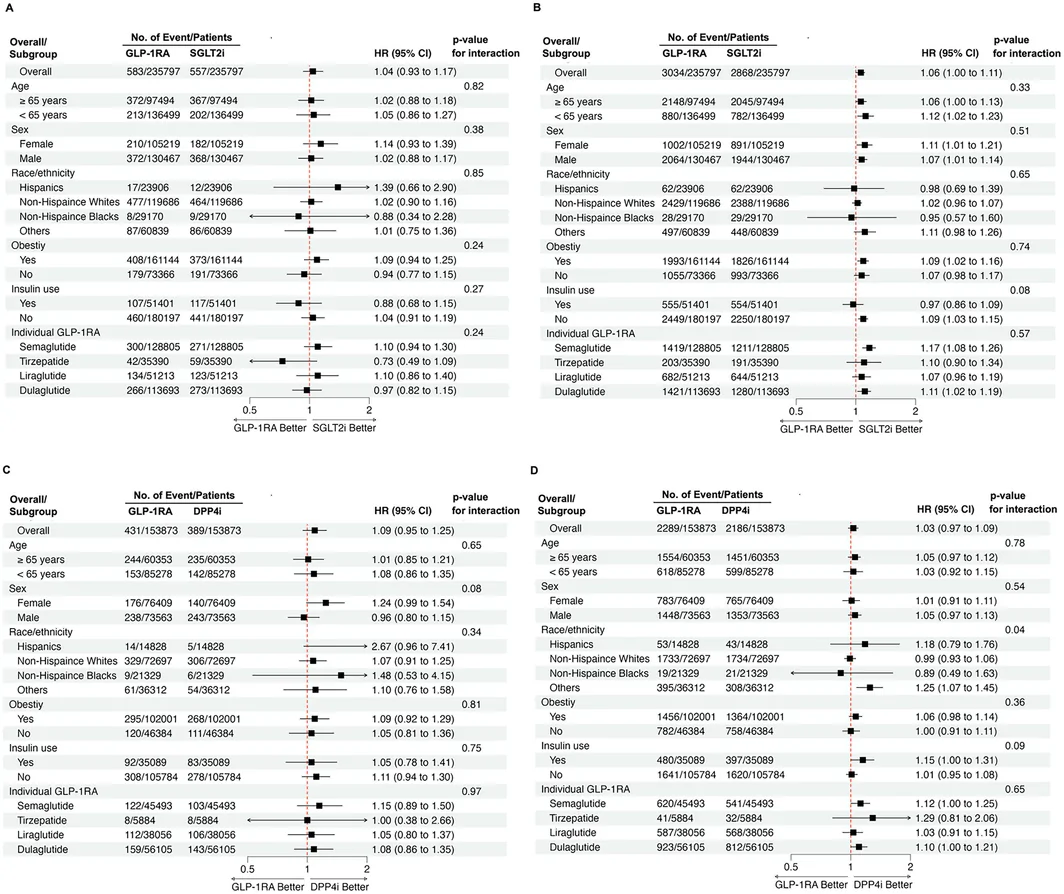
Subgroup analyses of the risk of melanoma and non‐melanom skin cancer in patients with type 2 diabetes initiating GLP‐1RA versus SGLT2i and GLP‐1RA versus DPP4i. (A) Melanoma (GLP‐1RA vs. SGLT2i). (B) Non‐melanoma skin cancer (GLP‐1RA vs. SGLT2i). (C) Melanoma (GLP‐1RA vs. DPP4i). (D) Non‐melanoma skin cancer (GLP‐1RA vs. DPP4i). CI, confidence interval; DPP4i, dipeptidyl peptidase‐4 inhibitor; GLP‐1RA, glucagon‐like peptide‐1 receptor agonist; HR, hazard ratio; SGLT2i, sodium–glucose cotransporter‐2 inhibitors.

**FIGURE 3:**
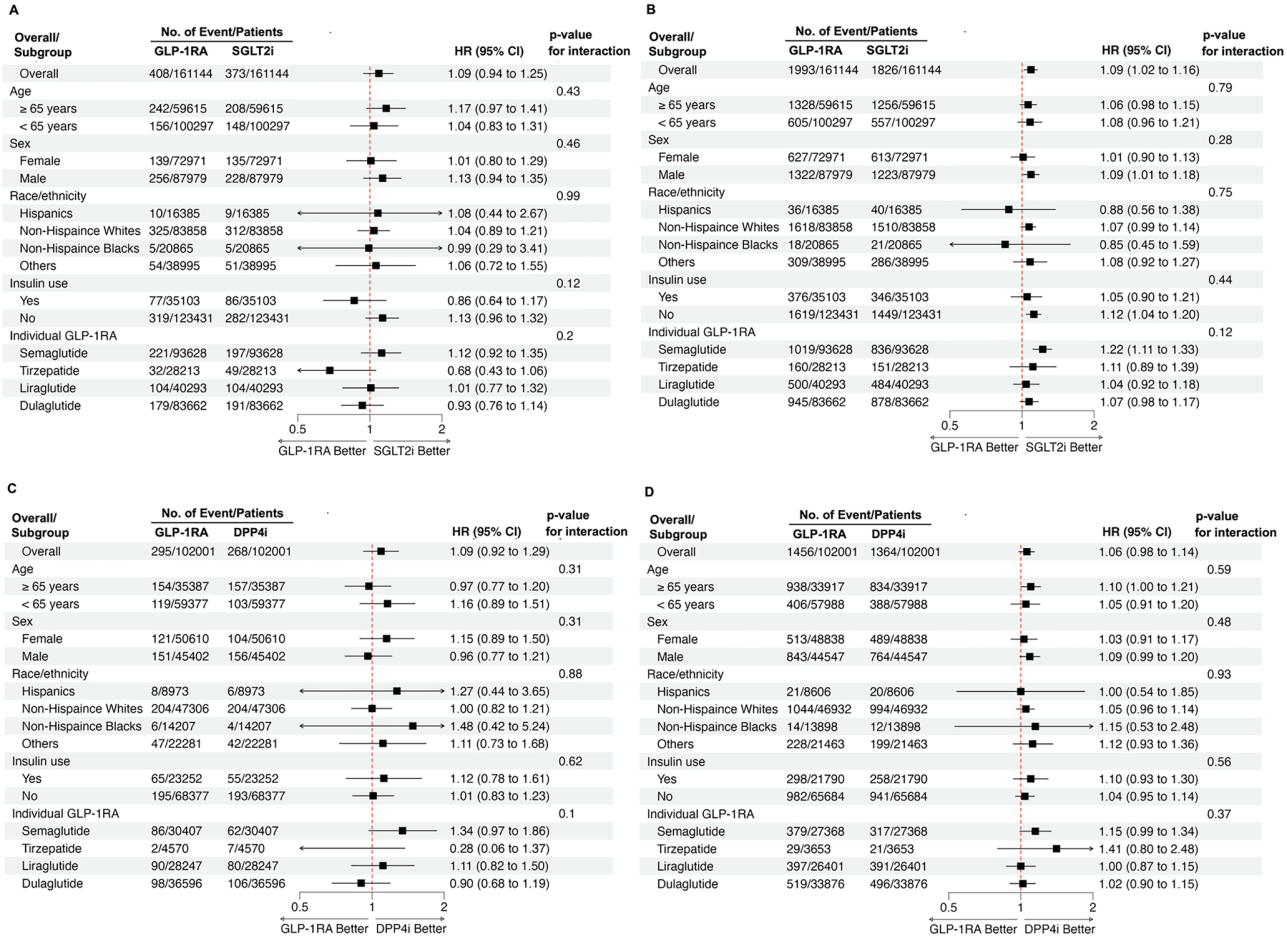
The risk of melanoma and non‐melanoma skin cancer in patients with type 2 diabetes and obesity initiating GLP‐1RA versus SGLT2i and GLP‐1RA versus DPP4i. (A) Melanoma (GLP‐1RA vs. SGLT2i). (B) Non‐melanoma skin cancer (GLP‐1RA vs. SGLT2i). (C) Melanoma (GLP‐1RAs vs. DPP4i). (D) Non‐melanoma skin cancer (GLP‐1RA vs. DPP4i). CI, confidence interval; DPP4i, dipeptidyl peptidase‐4 inhibitor; GLP‐1RA, glucagon‐like peptide‐1 receptor agonist; HR, hazard ratio; SGLT2i, sodium–glucose cotransporter‐2 inhibitors.

Across all examined subgroups (Figure 2), GLP‐1RA initiation was not significantly associated with an increased risk of melanoma compared with either SGLT2i or DPP4i, with no interaction reaching statistical significance (*p* > 0.05). For NMSC, associations were generally consistent across subgroups except for a significant interaction by race/ethnicity observed in the GLP‐1RA versus DPP4i comparison (*p* = 0.04). No significantly increased risk of NMSC was observed among Hispanic, non‐Hispanic White or non‐Hispanic Black patients; however, a higher risk was identified among patients categorised as other race/ethnicity (including Asian, American Indian or Alaska Native, Native Hawaiian or other Pacific Islander, other race or unknown) (HR, 1.25; 95% CI, 1.07–1.45). The results were consistent among patients with obesity (Figure 3).

## 4 Discussion

In this large, nationwide target trial emulation of adults with T2D, with over up to 3 years of follow‐up, GLP‐1RA initiation was not associated with an increased risk of melanoma compared with either SGLT2i or DPP4i. Although a borderline increase in NMSC risk was observed in the comparison with SGLT2is, no such association was identified in the comparison with DPP4is. The findings were robust in sensitivity analyses incorporating a 1‐year lag period and in analyses restricted to patients with obesity. Furthermore, the association with NMSC was attenuated after NCO calibration. Findings were generally consistent across prespecified subgroups.

These results should be interpreted in the context of prior evidence, which has been mixed. Early concerns regarding a potential association between GLP‐1RA use and skin cancer were largely driven by preclinical studies showing GLP‐1 receptor expression in melanocytes and keratinocytes and activation of potentially mitogenic signalling pathways [6, 10, 11]. In clinical trials, a signal of increased skin cancer incidence was reported in a large cardiovascular outcomes trial of liraglutide [12, 22]. However, cancer outcomes were not prespecified endpoints, event numbers were small and follow‐up duration was limited, restricting causal interpretation. Subsequent observational studies have reported inconsistent associations between GLP‐1RA use and melanoma or NMSC [4, 5, 23]. One real‐world study using US Department of Veterans Affairs databases observed a modestly increased risk of melanoma (HR, 1.15) and skin cancer (HR, 1.11) with GLP‐1RA use versus a composite group of sulfonylureas, DPP4is and SGLT2is, but no significant differences in head‐to‐head comparisons with DPP4is or SGLT2is^5^. In contrast, a population‐based cohort study using the United Kingdom Clinical Practice Research Datalink (2007–2019) found no significant difference between GLP‐1RA use and risk of melanoma (HR, 0.96) or NMSC (HR, 1.03) compared with sulfonylureas [23]. Similarly, an emulated trial using Danish nationwide health registries reported no association between GLP‐1RA and risk of melanoms compared with DPP4is (HR, 0.90) [4]. Prior studies, however, were limited by residual confounding related to obesity, diabetes severity or healthcare utilisation, which may partly explain inconsistent findings.

The present study extends the existing literature by addressing several of these limitations. By emulating a target trial using a new‐user, active‐comparator design and applying rigorous PSM, we minimised confounding by indication and baseline imbalances. In addition, NCO calibration strengthened causal inference by accounting for residual unmeasured confounding and systematic bias [24]. Overall, the results provide little evidence that GLP‐1RA use substantially increases the risk of melanoma or NMSC, although a slight increase in NMSC risk cannot be completely ruled out.

In this study, a borderline increase in NMSC risk was observed in the comparison of GLP‐1RAs versus SGLT2is but not versus DPP4is. Notably, this association was attenuated after NCO calibration, indicating that residual bias may have contributed to the observed signal. Given the small effect size, inconsistency across comparator groups and attenuation after calibration, the findings do not provide strong evidence for a causal association between GLP‐1RA use and NMSC risk. Furthermore, several subgroup findings warrant cautious consideration. Evidence of the effect heterogeneity by race and ethnicity was detected only in the comparison of GLP‐1RAs versus DPP4i and not elsewhere. This isolated finding may reflect differences in baseline NMSC risk or residual confounding from unmeasured socioeconomic or behavioural factors. Nonetheless, subgroup analyses are exploratory and susceptible to residual confounding, differential prescribing and multiple testing [25], warranting cautious interpretation and further investigation.

There are several limitations that warrant consideration. First, as with all observational studies using EHRs, residual confounding may persist despite extensive adjustment for demographic, clinical and health care utilisation factors. Important variables such as family history of skin cancer, cumulative ultraviolet radiation exposure, skin phototype, occupational sun exposure and socioeconomic factors may be incompletely captured or unavailable in the dataset [26]. Concomitant pharmacologic treatments during follow‐up may introduce residual confounding. Medications such as metformin, insulin, thiazolidinediones, corticosteroids and immunosuppressants may influence skin cancer risk and time‐varying use of these agents was not fully captured. Although baseline medication use was included in the propensity score models, treatment changes over time may still contribute to unmeasured confounding. Second, EHR data do not provide continuous enrollment information; therefore, patients may have differed in the duration of observable baseline history before treatment initiation. Consequently, some comorbidities and clinical characteristics may have been incompletely captured, potentially leading to residual confounding despite extensive covariate adjustment. Third, outcome definitions for melanoma and NMSC were based on ICD‐10‐CM diagnosis codes, with positive predictive values of approximately 58% for melanoma and 98% for NMSC [15], which may be subject to misclassification. Because downstream evidence such as surgery or chemotherapy was incompletely captured and TriNetX is not linked to cancer registries, incorporating such criteria could have reduced sensitivity and introduced selection bias. Therefore, diagnosis‐based definitions were used to ensure consistent ascertainment across sites. In addition, ICD codes do not reliably distinguish between basal cell carcinoma and squamous cell carcinoma, limiting evaluation of subtype‐specific risks. Fourth, missing data are an inherent limitation of EHR‐based studies. In this study, laboratory and vital sign variables were subject to substantial missingness and potential measurement bias due to differential testing practices across care settings, which may limit their comparability despite the use of imputation. Fifth, although the iIIT approach has advantages, including preservation of baseline comparability and reduced risk of time‐varying confounding, it may introduce exposure misclassification over follow‐up [27]. While we restricted follow‐up to 3 years to mitigate this concern, the inability to perform as‐treated or dose–response analyses due to incomplete prescription and adherence information remains an important limitation and may have resulted in residual exposure misclassification. Sixth, although NCOs were used as a sensitivity analysis to assess residual confounding, their validity depends on correct specification and reliable ascertainment in EHR data. Some outcomes may be poorly captured or not fully share the same confounding structure as the primary outcomes, which may limit calibration performance and introduce additional uncertainty. Finally, although follow‐up extended to 3 years, the mean follow‐up duration was approximately 1.8–2.2 years, which may be insufficient to detect skin cancer risks that manifest only after prolonged exposure or extended latency periods. This concern is particularly relevant for GLP‐1RAs, which are intended for long‐term use, and for newer agents such as semaglutide and tirzepatide, whose long‐term oncologic safety profiles have not yet been fully characterised.

## 5 Conclusions

In conclusion, GLP‐1RA use was not associated with increased risk of melanoma compared with SGLT2i or DPP4i. Although a modest increase in NMSC risk was observed in one active‐comparator analysis, the association was small, was not consistent across comparator groups and was attenuated after NCO calibration, suggesting that it may reflect residual bias rather than a causal effect. Overall, these results provide reassurance regarding the short term dermatologic safety of GLP‐1RAs, while highlight the need for continued surveillance and future studies examining longer‐term risk and medication‐specific effects.

## Funding

This work was supported in part by the National Institutes of Health (RF1AG077820, R01AG073435, R01DK128237).

## Conflicts of Interest

The authors declare no conflicts of interest.

## Supporting information


**Table S1:** Target trials emulation.
**Table S2:** Exposures of interest.
**Table S3:** Codes used for outcome definition and inclusion and exclusion criteria.
**Table S4:** List of 34 negative control outcomes.
**Table S5:** Baseline characteristics of patients in GLP‐1RA versus SGLT2i cohort before and after 1:1 propensity score matching (PSM).
**Table S6:** Baseline characteristics of patients in GLP‐1RA versus DPP4i cohort before and after 1:1 propensity score matching (PSM).
**Figure S1:** The flowchart of patient selection in TriNetX electronic health record data.
**Figure S2:** The propensity score distribution before and after propensity score matching.

## Data Availability

Data used in this study are available through the TriNetX research network: https://trinetx.com/.
