# Supplementary material for: GLP‐1 Receptor Agonists and Risk of Skin Cancer in Adults With Type 2 Diabetes: A Target Trial Emulation
Source: Diabetes Obes Metab. 2026 Jul 28;28(10):9377–84. doi: 10.1111/dom.71142 (PMC13538745; doi:10.1111/dom.71142)
Supplement: Supplementary file 1 [file DOM-28-9377-s001.docx]

**Supplementary materials**

**Supplement Table 1**. Target trials emulation

**Supplement Table 2**. Exposures of interest

**Supplement Table 3.** Codes used for outcome definition and inclusion and exclusion criteria

**Supplement Table 4**. List of 34 negative control outcomes

**Supplement Table 5.** Baseline characteristics of patients in GLP-1RA vs SGLT2i cohort before and after 1:1 propensity score matching (PSM)

**Supplement Table 6.** Baseline characteristics of patients in GLP-1RA vs DPP4i cohort before and after 1:1 propensity score matching (PSM)

**Supplement Figure 1.** The flowchart of patient selection in TriNetX electronic health record data

**Supplement Figure 2.** The propensity score distribution before and after propensity score matching.

**Supplement Table 1**. Target trial emulation

| **Approaches** | **Target trial** | **Target Trial Emulation** |
| --- | --- | --- |
| Eligibility criteria | - Type 2 diabetes - Age ≥18 years - No diagnosis of type 1 diabetes - No prescription for GLP-1RA, SGLT2i, or DPP4i within past year - No contraindication to study drugs (end-stage renal disease/dialysis) - No prior outcomes of interest | Same as for the target trial except that we required at least 1 encounter in the electronic health record system within one year before treatment initiation |
| Treatment strategies | Initiation treatment with a GLP-1RA, an SGLT2i, or a DPP4i | The date of initiation treatment was the date of the first prescription of a GLP-1RA, an SGLT2i, or a DPP4i |
| Treatment assignment | Individuals are randomly assigned to GLP-1RA, SGLT2i, or DPP4i at baseline. Individuals and their treating physicians will be aware of the assigned treatment strategy. | Treatment is not assigned randomly.  Randomization was emulated using 1:1 propensity score matching to address potential confounders. |
| Outcomes | Melanoma and non-melanoma skin cancer were defined using ICD-10 codes | Same as for the target trial |
| Follow-up | Follow-up for each individual started at treatment initiation and ended at the earliest of outcome onset, death, loss to follow-up, 3 years of follow-up, or study end (January 31, 2025). | Same as for the target trial. Loss to follow-up defined by last encounter date |
| Causal contrasts | Intention-to-treat effect | Observational analogs of the intention-to-treat |
| Statistical analysis | Cox proportional hazard regression models are used to estimate hazard ratios with 95% CIs by comparing participants assigned to GLP-1RAs versus SGLT2is and GLP-1RAs versus DPP4is. | Same as for the target trial  Negative control outcome (NCO) calibration was applied to address residual confounding |

GLP-1RAs, glucagon-like peptide-1 receptor agonists; DPP4i, dipeptidyl peptidase 4 inhibitors; SGLT2i, sodium-glucose cotransporter 2 inhibitors

**Supplement Table 2**. Exposures of interest

| **Classes** | **Medications** |
| --- | --- |
| GLP-1RA | Dulaglutide, Exenatide, Liraglutide, Lixisenatide, Semaglutide, Tirzepatide |
| SGLT2i | Canagliflozin, Dapagliflozin, Empagliflozin, Ertugliflozin, Sotagliflozin, Bexagliflozin |
| DPP4i | Sitagliptin, Saxagliptin, Alogliptin, Linagliptin |

GLP-1RA, glucagon-like peptide-1 receptor agonists; SGLT2i, sodium-glucose cotransporter 2 inhibitors; DPP4i, dipeptidyl peptidase 4 inhibitors.

**Supplement Table 3.** Codes used for outcome definition, inclusion, and exclusion criteria.

| **Outcome/Inclusion and exclusion criteria** | **ICD-10** |
| --- | --- |
| Type 2 diabetes | E11 |
| Type 1 diabetes | E10 |
| End-Stage Renal Disease /Dialysis | N18.5, N18.6, R88.0, T82.4, T85.611, T85.621, T85.631, T85.71, T86.1, Y84.1, Z48.22, Z49, Z94.0, Z99.2 |
| Melanoma | C43 |
| Non-melanoma skin cancer | C44 |

**Supplement Table 4**. List of 34 negative control outcomes

**List of negative control outcomes**

abnormal pupil

abrasion and friction burn of multiple sites

abrasion and friction burn of trunk without infection

acquired halluxvvalgus

acquired keratoderma

anal and rectal polyp

benign paroxysmal positional vertigo

burn of forearm

calcaneal spur

chondromalacia of patella

colostomy present

contact dermatitis

contusion of knee

derangement of knee

deviated nasal septum

epidermoid cyst

foreign body in ear

foreign body in orifice

ganglion cyst

hammer toe

impacted cerumen

ingrowing nail

lagophthalmos

lymphangioma

macular drusen

mechanical complication of internal orthopedic device implant graft

noise effects on inner ear

nontoxic multinodular goiter

presbyopia

regular astigmatism

sprain of ankle

strain of rotator cuff capsule

tear film insufficiency

tooth loss

**Supplement Table 5.** Baseline characteristics of patients in GLP-1RA vs SGLT2i cohort before and after 1:1 propensity score matching (PSM)^a^

|  | **Before PSM** | | | **After PSM** | | |
| --- | --- | --- | --- | --- | --- | --- |
|  | GLP-1RA | SGLT2i | SMD | GLP-1RA | SGLT2i | SMD |
| n | 407709 | 316488 |  | 235797 | 235797 |  |
| Age, yrs, mean(sd) | 57.33 (12.86) | 63.18 (12.39) | 0.463 | 60.61 (11.99) | 61.24 (12.27) | 0.052 |
| Age ≥ 65 yrs | 128149 (31.4) | 154432 (48.8) | 0.36 | 95481 (40.5) | 99922 (42.4) | 0.038 |
| Female | 228890 (56.1) | 125910 (39.8) | 0.332 | 109132 (46.3) | 104751 (44.4) | 0.037 |
| **Race/ethnicity** | | | | | | |
| Hispanics | 39510 (9.7) | 30919 (9.8) | 0.078 | 23966 (10.2) | 23987 (10.2) | 0.007 |
| Non-Hispanic Whites | 209017 (51.3) | 154597 (48.8) |  | 119258 (50.6) | 118616 (50.3) |  |
| Non-Hispanic Blacks | 58078 (14.2) | 42000 (13.3) |  | 30669 (13.0) | 30588 (13.0) |  |
| Others/unknown | 101104 (24.8) | 88972 (28.1) |  | 61904 (26.3) | 62606 (26.6) |  |
| **Entry year** | | | | | | |
| 2014 | 2737 (0.7) | 1754 (0.6) | 0.111 | 1753 (0.7) | 1654 (0.7) | 0.013 |
| 2015 | 5267 (1.3) | 4857 (1.5) |  | 4084 (1.7) | 4255 (1.8) |  |
| 2016 | 11294 (2.8) | 9884 (3.1) |  | 8357 (3.5) | 8506 (3.6) |  |
| 2017 | 14041 (3.4) | 11146 (3.5) |  | 9639 (4.1) | 9683 (4.1) |  |
| 2018 | 19977 (4.9) | 13698 (4.3) |  | 12421 (5.3) | 12175 (5.2) |  |
| 2019 | 25506 (6.3) | 19791 (6.3) |  | 16794 (7.1) | 16722 (7.1) |  |
| 2020 | 27256 (6.7) | 24846 (7.9) |  | 18899 (8.0) | 19133 (8.1) |  |
| 2021 | 47272 (11.6) | 41531 (13.1) |  | 30416 (12.9) | 30703 (13.0) |  |
| 2022 | 67929 (16.7) | 57736 (18.2) |  | 40202 (17.0) | 40485 (17.2) |  |
| 2023 | 98340 (24.1) | 65945 (20.8) |  | 48341 (20.5) | 47807 (20.3) |  |
| 2024 | 85059 (20.9) | 62882 (19.9) |  | 43273 (18.4) | 42992 (18.2) |  |
| 2025 | 3031 (0.7) | 2418 (0.8) |  | 1618 (0.7) | 1682 (0.7) |  |
| **Marrital status** |  |  | 0.028 |  |  | 0.006 |
| Married | 143105 (35.1) | 109602 (34.6) | 0.028 | 83840 (35.6) | 84293 (35.7) | 0.006 |
| Single | 107022 (26.2) | 80398 (25.4) |  | 59417 (25.2) | 58800 (24.9) |  |
| Others/unknown | 157582 (38.7) | 126488 (40.0) |  | 92540 (39.2) | 92704 (39.3) |  |
| **Region** |  |  | 0.218 |  |  | 0.016 |
| Northeast | 105177 (25.8) | 80985 (25.6) |  | 62304 (26.4) | 62218 (26.4) |  |
| South | 163253 (40.0) | 121497 (38.4) |  | 92428 (39.2) | 91717 (38.9) |  |
| Midwest | 78434 (19.2) | 53745 (17.0) |  | 41955 (17.8) | 41891 (17.8) |  |
| West | 58651 (14.4) | 49382 (15.6) |  | 36932 (15.7) | 37461 (15.9) |  |
| Others/unknown | 2194 (0.5) | 10879 (3.4) |  | 2178 (0.9) | 2510 (1.1) |  |
| **No. of encounters** | | | | | | |
| ≤ 5 | 182850 (44.8) | 140756 (44.5) | 0.041 | 109270 (46.3) | 110092 (46.7) | 0.007 |
| >5 and ≤ 10 | 88113 (21.6) | 66084 (20.9) |  | 50788 (21.5) | 50526 (21.4) |  |
| >10 and ≤ 20 | 74764 (18.3) | 56964 (18.0) |  | 41913 (17.8) | 41434 (17.6) |  |
| > 20 | 61982 (15.2) | 52684 (16.6) |  | 33826 (14.3) | 33745 (14.3) |  |
| **No. of emergency/hospital visits** | | | | | | |
| 0 | 359256 (88.1) | 232440 (73.4) | 0.379 | 199416 (84.6) | 197294 (83.7) | 0.025 |
| ≥1 and ≤ 5 | 47210 (11.6) | 81152 (25.6) |  | 35427 (15.0) | 37476 (15.9) |  |
| > 5 | 1243 (0.3) | 2896 (0.9) |  | 954 (0.4) | 1027 (0.4) |  |
| **Comorbidities** | | | | | | |
| Diabetic nephropathy | 36889 (9.0) | 50383 (15.9) | 0.209 | 27289 (11.6) | 28891 (12.3) | 0.021 |
| Diabetic neuropathy | 43048 (10.6) | 36317 (11.5) | 0.029 | 26452 (11.2) | 26294 (11.2) | 0.002 |
| Diabetic retinopathy | 14316 (3.5) | 11799 (3.7) | 0.012 | 8989 (3.8) | 8915 (3.8) | 0.002 |
| Ischemic heart disease | 41312 (10.1) | 84990 (26.9) | 0.441 | 34697 (14.7) | 37309 (15.8) | 0.031 |
| Heart failure | 20871 (5.1) | 73908 (23.4) | 0.541 | 20172 (8.6) | 22348 (9.5) | 0.032 |
| Cerebrovascular disease | 12412 (3.0) | 21178 (6.7) | 0.17 | 9686 (4.1) | 10360 (4.4) | 0.014 |
| Peripheral vascular disease | 17248 (4.2) | 33692 (10.6) | 0.246 | 13809 (5.9) | 14696 (6.2) | 0.016 |
| Hypertension | 231917 (56.9) | 212412 (67.1) | 0.212 | 143810 (61.0) | 145946 (61.9) | 0.019 |
| Hyperlipidemia | 205294 (50.4) | 181730 (57.4) | 0.142 | 125512 (53.2) | 127062 (53.9) | 0.013 |
| Chronic kidney disease | 35687 (8.8) | 56211 (17.8) | 0.268 | 28037 (11.9) | 30146 (12.8) | 0.027 |
| Obesity | 320005 (78.5) | 196367 (62.0) | 0.366 | 163941 (69.5) | 159467 (67.6) | 0.041 |
| Alzheimer's disease and related dementias | 2414 (0.6) | 6004 (1.9) | 0.118 | 2032 (0.9) | 2291 (1.0) | 0.012 |
| Epilepsy/seizures | 2689 (0.7) | 3237 (1.0) | 0.04 | 1681 (0.7) | 1757 (0.7) | 0.004 |
| Sleep disorders | 68505 (16.8) | 47761 (15.1) | 0.047 | 34189 (14.5) | 33324 (14.1) | 0.01 |
| Depression | 48912 (12.0) | 30072 (9.5) | 0.081 | 23107 (9.8) | 22190 (9.4) | 0.013 |
| Bipolar disorder | 6658 (1.6) | 4096 (1.3) | 0.028 | 3192 (1.4) | 3054 (1.3) | 0.005 |
| Anxiety | 47344 (11.6) | 29345 (9.3) | 0.077 | 22208 (9.4) | 21260 (9.0) | 0.014 |
| Glaucoma | 7104 (1.7) | 6634 (2.1) | 0.026 | 4481 (1.9) | 4649 (2.0) | 0.005 |
| Cataract | 10599 (2.6) | 8323 (2.6) | 0.002 | 6390 (2.7) | 6495 (2.8) | 0.003 |
| Asthma | 26816 (6.6) | 16415 (5.2) | 0.059 | 12635 (5.4) | 12215 (5.2) | 0.008 |
| Thyroid disease | 55571 (13.6) | 37882 (12.0) | 0.05 | 28752 (12.2) | 28101 (11.9) | 0.008 |
| Anemia | 31211 (7.7) | 41352 (13.1) | 0.178 | 20234 (8.6) | 21253 (9.0) | 0.015 |
| Pneumonia | 5215 (1.3) | 11637 (3.7) | 0.155 | 3964 (1.7) | 4282 (1.8) | 0.01 |
| Chronic obstructive pulmonary disease | 19186 (4.7) | 27509 (8.7) | 0.16 | 13047 (5.5) | 13631 (5.8) | 0.011 |
| Other substance use disorders | 26840 (6.6) | 32980 (10.4) | 0.138 | 17666 (7.5) | 18042 (7.7) | 0.006 |
| Osteoporosis | 5731 (1.4) | 5780 (1.8) | 0.033 | 3812 (1.6) | 3879 (1.6) | 0.002 |
| Rheumatoid arthritis/osteoarthritis | 4917 (1.2) | 3917 (1.2) | 0.003 | 2663 (1.1) | 2657 (1.1) | <0.001 |
| Inflammatory bowel disease | 2073 (0.5) | 1707 (0.5) | 0.004 | 1207 (0.5) | 1199 (0.5) | <0.001 |
| Acute pancreatitis | 672 (0.2) | 1661 (0.5) | 0.061 | 616 (0.3) | 724 (0.3) | 0.009 |
| HIV/AIDS | 2357 (0.6) | 1747 (0.6) | 0.003 | 1326 (0.6) | 1337 (0.6) | 0.001 |
| Cancer | 18620 (4.6) | 20293 (6.4) | 0.081 | 12436 (5.3) | 12890 (5.5) | 0.009 |
| Chronic pain | 38998 (9.6) | 28230 (8.9) | 0.022 | 20714 (8.8) | 20385 (8.6) | 0.005 |
| Persons with socioeconomic health risks | 4574 (1.1) | 5836 (1.8) | 0.06 | 2788 (1.2) | 2836 (1.2) | 0.002 |
| Problems related to lifestyle | 8529 (2.1) | 8638 (2.7) | 0.042 | 5115 (2.2) | 5134 (2.2) | 0.001 |
| **Medications** | | | | | | |
| Metformin | 124326 (30.5) | 97284 (30.7) | 0.005 | 77092 (32.7) | 77628 (32.9) | 0.005 |
| Insulin | 80746 (19.8) | 85339 (27.0) | 0.17 | 52187 (22.1) | 52317 (22.2) | 0.001 |
| Sulfonylureas | 32523 (8.0) | 34446 (10.9) | 0.1 | 24339 (10.3) | 25435 (10.8) | 0.015 |
| Thiazolidinediones | 7003 (1.7) | 7601 (2.4) | 0.048 | 5313 (2.3) | 5490 (2.3) | 0.005 |
| DPP4is | 462 (0.1) | 580 (0.2) | 0.018 | 373 (0.2) | 395 (0.2) | 0.002 |
| ACEis | 62664 (15.4) | 64339 (20.3) | 0.13 | 43551 (18.5) | 44831 (19.0) | 0.014 |
| Angiotensin-receptor blockers | 53358 (13.1) | 70231 (22.2) | 0.241 | 38380 (16.3) | 39578 (16.8) | 0.014 |
| Diuretics | 54883 (13.5) | 80003 (25.3) | 0.302 | 38903 (16.5) | 40496 (17.2) | 0.018 |
| Beta blockers | 60326 (14.8) | 98231 (31.0) | 0.394 | 46512 (19.7) | 49466 (21.0) | 0.031 |
| Calcium channel blockers | 45494 (11.2) | 53763 (17.0) | 0.168 | 32279 (13.7) | 33738 (14.3) | 0.018 |
| Aspirin | 26361 (6.5) | 59367 (18.8) | 0.377 | 22750 (9.6) | 24875 (10.5) | 0.03 |
| Antidepressants | 64246 (15.8) | 48263 (15.2) | 0.014 | 35031 (14.9) | 34455 (14.6) | 0.007 |
| NSAIDs | 45667 (11.2) | 33493 (10.6) | 0.02 | 25826 (11.0) | 25704 (10.9) | 0.002 |
| Proton pump inhibitors | 48967 (12.0) | 54940 (17.4) | 0.152 | 32099 (13.6) | 32843 (13.9) | 0.009 |
| Opioid | 121134 (29.7) | 142034 (44.9) | 0.318 | 86654 (36.7) | 89757 (38.1) | 0.027 |
| Statins | 48877 (12.0) | 44400 (14.0) | 0.061 | 29008 (12.3) | 29188 (12.4) | 0.002 |
| Corticosteroids | 5196 (1.3) | 4203 (1.3) | 0.005 | 3122 (1.3) | 3094 (1.3) | 0.001 |
| Immunosuppressants | 102903 (25.2) | 99733 (31.5) | 0.139 | 63732 (27.0) | 64653 (27.4) | 0.009 |
| Anti-obesity drugs | 14658 (3.6) | 4011 (1.3) | 0.152 | 4439 (1.9) | 3673 (1.6) | 0.025 |
| **Lab and vital values^b^** | | | | | | |
| **After imputation** | | | | | | |
| HbA1c, %, mean(sd) | 8.08 (1.89) | 8.08 (1.80) | 0.004 | 8.20 (1.90) | 8.19 (1.79) | 0.004 |
| BMI, kg/m^2^, mean(sd) | 34.89 (6.39) | 32.17 (6.41) | 0.424 | 33.32 (6.31) | 33.01 (6.35) | 0.05 |
| Systolic blood pressure, mmHg, mean(sd) | 130.74 (15.97) | 130.35 (17.19) | 0.023 | 130.97 (16.31) | 131.03 (16.70) | 0.003 |
| Diastolic blood pressure, mmHg, mean(sd) | 77.26 (10.11) | 75.23 (10.76) | 0.194 | 76.36 (10.23) | 76.18 (10.44) | 0.017 |
| eGFR, mL/min/1.73m², mean(sd) | 83.77 (26.59) | 76.90 (26.92) | 0.257 | 80.45 (26.62) | 79.78 (26.66) | 0.025 |
| High-density lipoprotein cholesterol, mg/dL, mean(sd) | 42.68 (15.99) | 41.62 (16.03) | 0.067 | 42.24 (15.95) | 42.22 (16.07) | 0.002 |
| Low-density lipoprotein cholesterol, mg/dL, mean(sd) | 93.41 (36.85) | 89.38 (36.85) | 0.11 | 91.10 (36.71) | 90.81 (36.87) | 0.008 |
| Total cholesterol, mg/dL, mean(sd) | 171.45 (43.42) | 164.88 (44.01) | 0.15 | 168.59 (43.53) | 168.10 (43.68) | 0.011 |
| Triglycerides, mg/dL, mean(sd) | 178.30 (109.21) | 170.28 (107.52) | 0.074 | 178.07 (109.73) | 177.24 (110.46) | 0.008 |
| **Before Imputation^c^** | | | | | | |
| HbA1c, %, mean(sd) | 8.07 (1.93) | 8.17 (1.78) | 0.052 | - | - | - |
| BMI, kg/m^2^, mean(sd) | 35.40 (6.20) | 31.77 (6.27) | 0.583 | - | - | - |
| Systolic blood pressure, mmHg, mean(sd) | 130.83 (15.55) | 130.16 (17.31) | 0.041 | - | - | - |
| Diastolic blood pressure, mmHg, mean(sd) | 77.40 (9.88) | 75.02 (10.82) | 0.23 | - | - | - |
| eGFR, mL/min/1.73m², mean(sd) | 81.86 (26.83) | 73.98 (27.06) | 0.292 | - | - | - |
| High-density lipoprotein cholesterol, mg/dL, mean(sd) | 41.57 (15.22) | 40.13 (15.49) | 0.094 | - | - | - |
| Low-density lipoprotein cholesterol, mg/dL, mean(sd) | 94.93 (36.12) | 86.89 (35.90) | 0.223 | - | - | - |
| Total cholesterol, mg/dL, mean(sd) | 173.53 (43.84) | 163.15 (44.91) | 0.234 | - | - | - |
| Triglycerides, mg/dL, mean(sd) | 180.93 (110.23) | 173.89 (111.82) | 0.063 | - | - | - |

^a^ Values are numbers (percentages) unless otherwise indicated.

^b^ Missing data were imputed using multiple imputation by chained equations, including all baseline covariates

^c^ The proportion of missing values for HbA1c, BMI, systolic blood pressure, diastolic blood pressure, eGFR, High-density lipoprotein cholesterol, Low-density lipoprotein cholesterol, Total cholesterol, Triglycerides were 41.7%, 52.8%, 33.6%, 33.6%, 49.1%, 60.7%,63.9%,61.9%, and 62.1%.

Abbreviations: GLP-1RA, glucagon-like peptide-1 receptor agonist; SGLT2is, sodium–glucose cotransporter-2 inhibitors; DPP4is, dipeptidyl peptidase-4 inhibitor; SMD, standardized mean difference; ACEi, angiotensin-converting enzyme inhibitor; BMI, body mass index; NSAIDs, nonsteroidal anti-inflammatory drugs; HIV/AIDS, human immunodeficiency virus/acquired immunodeficiency syndrome; eGFR, estimated glomerular filtration rate

**Supplement Table 6.** Baseline characteristics of patients in GLP-1RA vs DPP4i cohort before and after 1:1 propensity score matching (PSM)^a^

|  | **Before PSM** | | | **After PSM** | | |
| --- | --- | --- | --- | --- | --- | --- |
|  | GLP-1RA | DPP4i | SMD | GLP-1RA | DPP4i | SMD |
| n | 408393 | 239701 |  | 153873 | 153873 |  |
| Age, yrs, mean(sd) | 57.34 (12.86) | 63.69 (12.51) | 0.501 | 60.90 (12.04) | 61.41 (12.92) | 0.041 |
| Age ≥ 65 yrs | 128357 (31.4) | 122203 (51.0) | 0.405 | 64537 (41.9) | 66207 (43.0) | 0.022 |
| Female | 229191 (56.1) | 114847 (47.9) | 0.165 | 78330 (50.9) | 77527 (50.4) | 0.01 |
| **Race/ethnicity** | | | | | | |
| Hispanics | 39561 (9.7) | 24900 (10.4) | 0.121 | 16038 (10.4) | 16052 (10.4) | 0.011 |
| Non-Hispanic Whites | 209321 (51.3) | 109327 (45.6) |  | 75695 (49.2) | 75024 (48.8) |  |
| Non-Hispanic Blacks | 58134 (14.2) | 35348 (14.7) |  | 22679 (14.7) | 22666 (14.7) |  |
| Others/unknown | 101377 (24.8) | 70126 (29.3) |  | 39461 (25.6) | 40131 (26.1) |  |
| **Entry year** | | | | | | |
| 2014 | 2728 (0.7) | 9401 (3.9) | 1.056 | 2699 (1.8) | 3102 (2.0) | 0.057 |
| 2015 | 5237 (1.3) | 15454 (6.4) |  | 5094 (3.3) | 5690 (3.7) |  |
| 2016 | 11314 (2.8) | 29429 (12.3) |  | 10883 (7.1) | 12002 (7.8) |  |
| 2017 | 14045 (3.4) | 28892 (12.1) |  | 12971 (8.4) | 14017 (9.1) |  |
| 2018 | 19995 (4.9) | 28996 (12.1) |  | 16742 (10.9) | 17196 (11.2) |  |
| 2019 | 25527 (6.3) | 28405 (11.9) |  | 19159 (12.5) | 19146 (12.4) |  |
| 2020 | 27272 (6.7) | 24168 (10.1) |  | 17721 (11.5) | 17133 (11.1) |  |
| 2021 | 47350 (11.6) | 23941 (10.0) |  | 20518 (13.3) | 19412 (12.6) |  |
| 2022 | 68105 (16.7) | 20792 (8.7) |  | 18631 (12.1) | 18036 (11.7) |  |
| 2023 | 98537 (24.1) | 17622 (7.4) |  | 16663 (10.8) | 16204 (10.5) |  |
| 2024 | 85250 (20.9) | 12137 (5.1) |  | 12340 (8.0) | 11495 (7.5) |  |
| 2025 | 3033 (0.7) | 464 (0.2) |  | 452 (0.3) | 440 (0.3) |  |
| **Marrital status** |  |  | 0.047 |  |  | 0.009 |
| Married | 143344 (35.1) | 78816 (32.9) | 0.047 | 51501 (33.5) | 52097 (33.9) | 0.009 |
| Single | 107173 (26.2) | 64862 (27.1) |  | 41180 (26.8) | 41203 (26.8) |  |
| Others/unknown | 157876 (38.7) | 96023 (40.1) |  | 61192 (39.8) | 60573 (39.4) |  |
| **Region** |  |  | 0.297 |  |  | 0.036 |
| Northeast | 105308 (25.8) | 58178 (24.3) |  | 40514 (26.3) | 39748 (25.8) |  |
| South | 163586 (40.1) | 98672 (41.2) |  | 63328 (41.2) | 64345 (41.8) |  |
| Midwest | 78493 (19.2) | 36012 (15.0) |  | 26099 (17.0) | 25227 (16.4) |  |
| West | 58811 (14.4) | 34493 (14.4) |  | 21778 (14.2) | 21817 (14.2) |  |
| Others/unknown | 2195 (0.5) | 12346 (5.2) |  | 2154 (1.4) | 2736 (1.8) |  |
| **No. of encounters** | | | | | | |
| ≤ 5 | 183110 (44.8) | 111289 (46.4) | 0.035 | 69625 (45.2) | 70594 (45.9) | 0.014 |
| >5 and ≤ 10 | 88279 (21.6) | 50803 (21.2) |  | 33131 (21.5) | 33125 (21.5) |  |
| >10 and ≤ 20 | 74896 (18.3) | 41533 (17.3) |  | 27660 (18.0) | 27192 (17.7) |  |
| > 20 | 62108 (15.2) | 36076 (15.1) |  | 23457 (15.2) | 22962 (14.9) |  |
| **No. of emergency/hospital visits** | | | | | | |
| 0 | 359844 (88.1) | 181664 (75.8) | 0.325 | 125560 (81.6) | 124285 (80.8) | 0.021 |
| ≥1 and ≤ 5 | 47301 (11.6) | 56193 (23.4) |  | 27505 (17.9) | 28709 (18.7) |  |
| > 5 | 1248 (0.3) | 1844 (0.8) |  | 808 (0.5) | 879 (0.6) |  |
| **Comorbidities** | | | | | | |
| Diabetic nephropathy | 36965 (9.1) | 35167 (14.7) | 0.174 | 18713 (12.2) | 19168 (12.5) | 0.009 |
| Diabetic neuropathy | 43102 (10.6) | 28008 (11.7) | 0.036 | 19305 (12.5) | 19017 (12.4) | 0.006 |
| Diabetic retinopathy | 14350 (3.5) | 9449 (3.9) | 0.023 | 6447 (4.2) | 6319 (4.1) | 0.004 |
| Ischemic heart disease | 41449 (10.1) | 41095 (17.1) | 0.205 | 20584 (13.4) | 21453 (13.9) | 0.016 |
| Heart failure | 21007 (5.1) | 23085 (9.6) | 0.172 | 11013 (7.2) | 11514 (7.5) | 0.013 |
| Cerebrovascular disease | 12436 (3.0) | 16275 (6.8) | 0.174 | 7117 (4.6) | 7472 (4.9) | 0.011 |
| Peripheral vascular disease | 17304 (4.2) | 16729 (7.0) | 0.119 | 8629 (5.6) | 8958 (5.8) | 0.009 |
| Hypertension | 232339 (56.9) | 155184 (64.7) | 0.161 | 93684 (60.9) | 95008 (61.7) | 0.018 |
| Hyperlipidemia | 205685 (50.4) | 128690 (53.7) | 0.067 | 80235 (52.1) | 80885 (52.6) | 0.008 |
| Chronic kidney disease | 35768 (8.8) | 39299 (16.4) | 0.232 | 19415 (12.6) | 20059 (13.0) | 0.013 |
| Obesity | 320513 (78.5) | 142948 (59.6) | 0.416 | 105303 (68.4) | 104043 (67.6) | 0.018 |
| Alzheimer's disease and related dementias | 2417 (0.6) | 7132 (3.0) | 0.181 | 1985 (1.3) | 2241 (1.5) | 0.014 |
| Epilepsy/seizures | 2693 (0.7) | 2801 (1.2) | 0.054 | 1323 (0.9) | 1360 (0.9) | 0.003 |
| Sleep disorders | 68648 (16.8) | 27279 (11.4) | 0.156 | 20662 (13.4) | 19998 (13.0) | 0.013 |
| Depression | 48962 (12.0) | 22628 (9.4) | 0.082 | 16057 (10.4) | 15703 (10.2) | 0.008 |
| Bipolar disorder | 6665 (1.6) | 3339 (1.4) | 0.02 | 2338 (1.5) | 2249 (1.5) | 0.005 |
| Anxiety | 47403 (11.6) | 20460 (8.5) | 0.102 | 14540 (9.4) | 14190 (9.2) | 0.008 |
| Glaucoma | 7121 (1.7) | 5920 (2.5) | 0.051 | 3276 (2.1) | 3370 (2.2) | 0.004 |
| Cataract | 10623 (2.6) | 7176 (3.0) | 0.024 | 4487 (2.9) | 4550 (3.0) | 0.002 |
| Asthma | 26850 (6.6) | 12422 (5.2) | 0.059 | 8677 (5.6) | 8491 (5.5) | 0.005 |
| Thyroid disease | 55665 (13.6) | 30328 (12.7) | 0.029 | 20026 (13.0) | 20063 (13.0) | 0.001 |
| Anemia | 31265 (7.7) | 33230 (13.9) | 0.201 | 15739 (10.2) | 16467 (10.7) | 0.015 |
| Pneumonia | 5229 (1.3) | 8916 (3.7) | 0.157 | 3309 (2.2) | 3567 (2.3) | 0.011 |
| Chronic obstructive pulmonary disease | 19224 (4.7) | 17355 (7.2) | 0.107 | 9085 (5.9) | 9462 (6.1) | 0.01 |
| Other substance use disorders | 26891 (6.6) | 19936 (8.3) | 0.066 | 11766 (7.6) | 11977 (7.8) | 0.005 |
| Osteoporosis | 5737 (1.4) | 5226 (2.2) | 0.058 | 2697 (1.8) | 2840 (1.8) | 0.007 |
| Rheumatoid arthritis/osteoarthritis | 4925 (1.2) | 2889 (1.2) | <0.001 | 1718 (1.1) | 1818 (1.2) | 0.006 |
| Inflammatory bowel disease | 2075 (0.5) | 1251 (0.5) | 0.002 | 782 (0.5) | 812 (0.5) | 0.003 |
| Acute pancreatitis | 672 (0.2) | 890 (0.4) | 0.04 | 376 (0.2) | 386 (0.3) | 0.001 |
| HIV/AIDS | 2361 (0.6) | 1352 (0.6) | 0.002 | 947 (0.6) | 946 (0.6) | <0.001 |
| Cancer | 18652 (4.6) | 18048 (7.5) | 0.125 | 8877 (5.8) | 9127 (5.9) | 0.007 |
| Chronic pain | 39046 (9.6) | 19428 (8.1) | 0.051 | 13727 (8.9) | 13521 (8.8) | 0.005 |
| Persons with socioeconomic health risks | 4584 (1.1) | 2810 (1.2) | 0.005 | 1793 (1.2) | 1782 (1.2) | 0.001 |
| Problems related to lifestyle | 8542 (2.1) | 5230 (2.2) | 0.006 | 3352 (2.2) | 3356 (2.2) | <0.001 |
| **Medications** | | | | | | |
| Metformin | 124519 (30.5) | 78390 (32.7) | 0.048 | 51982 (33.8) | 51971 (33.8) | <0.001 |
| Insulin | 80912 (19.8) | 70825 (29.5) | 0.227 | 41743 (27.1) | 41552 (27.0) | 0.003 |
| Sulfonylureas | 32542 (8.0) | 43674 (18.2) | 0.307 | 21086 (13.7) | 21998 (14.3) | 0.017 |
| Thiazolidinediones | 7028 (1.7) | 7474 (3.1) | 0.091 | 4078 (2.7) | 4196 (2.7) | 0.005 |
| SGLT2is | 0.00 (0.00) | 0.00 (0.00) | <0.001 | 398 (0.3) | 356 (0.2) | 0.006 |
| ACEis | 62790 (15.4) | 54855 (22.9) | 0.192 | 31312 (20.3) | 32177 (20.9) | 0.014 |
| Angiotensin-receptor blockers | 53551 (13.1) | 40490 (16.9) | 0.106 | 23518 (15.3) | 23988 (15.6) | 0.008 |
| Diuretics | 55038 (13.5) | 47478 (19.8) | 0.171 | 26030 (16.9) | 26800 (17.4) | 0.013 |
| Beta blockers | 60548 (14.8) | 65394 (27.3) | 0.309 | 32800 (21.3) | 34183 (22.2) | 0.022 |
| Calcium channel blockers | 45591 (11.2) | 46789 (19.5) | 0.233 | 23214 (15.1) | 24299 (15.8) | 0.02 |
| Aspirin | 26436 (6.5) | 42434 (17.7) | 0.35 | 18322 (11.9) | 19504 (12.7) | 0.023 |
| Antidepressants | 64387 (15.8) | 38028 (15.9) | 0.003 | 25093 (16.3) | 24907 (16.2) | 0.003 |
| NSAIDs | 45785 (11.2) | 29734 (12.4) | 0.037 | 19197 (12.5) | 19376 (12.6) | 0.004 |
| Proton pump inhibitors | 49108 (12.0) | 45954 (19.2) | 0.198 | 24308 (15.8) | 25094 (16.3) | 0.014 |
| Opioid | 121474 (29.7) | 104311 (43.5) | 0.289 | 58230 (37.8) | 59585 (38.7) | 0.018 |
| Statins | 48982 (12.0) | 36587 (15.3) | 0.095 | 21201 (13.8) | 21780 (14.2) | 0.011 |
| Corticosteroids | 5201 (1.3) | 4636 (1.9) | 0.053 | 2621 (1.7) | 2641 (1.7) | 0.001 |
| Immunosuppressants | 103079 (25.2) | 83231 (34.7) | 0.208 | 47908 (31.1) | 48804 (31.7) | 0.013 |
| Anti-obesity drugs | 14684 (3.6) | 2829 (1.2) | 0.159 | 3395 (2.2) | 2540 (1.7) | 0.04 |
| **Lab and vital values^b^** | | | | | | |
| **After imputation** | | | | | | |
| HbA1c, %, mean(sd) | 8.08 (1.89) | 8.26 (1.82) | 0.1 | 8.33 (1.91) | 8.31 (1.82) | 0.014 |
| BMI, kg/m^2^, mean(sd) | 34.89 (6.39) | 31.91 (6.44) | 0.463 | 33.24 (6.39) | 33.05 (6.49) | 0.03 |
| Systolic blood pressure, mmHg, mean(sd) | 130.74 (15.97) | 131.61 (17.27) | 0.052 | 131.32 (16.54) | 131.35 (16.93) | 0.002 |
| Diastolic blood pressure, mmHg, mean(sd) | 77.25 (10.11) | 74.78 (10.72) | 0.237 | 75.83 (10.33) | 75.69 (10.58) | 0.014 |
| eGFR, mL/min/1.73m², mean(sd) | 83.76 (26.59) | 74.89 (28.52) | 0.322 | 78.64 (26.89) | 78.07 (28.41) | 0.021 |
| High-density lipoprotein cholesterol, mg/dL, mean(sd) | 42.68 (15.99) | 42.63 (16.46) | 0.003 | 42.38 (16.05) | 42.43 (16.45) | 0.003 |
| Low-density lipoprotein cholesterol, mg/dL, mean(sd) | 93.41 (36.85) | 90.35 (36.59) | 0.083 | 91.25 (36.73) | 91.06 (36.69) | 0.005 |
| Total cholesterol, mg/dL, mean(sd) | 171.44 (43.42) | 167.28 (43.51) | 0.096 | 169.02 (43.51) | 168.78 (43.47) | 0.006 |
| Triglycerides, mg/dL, mean(sd) | 178.29 (109.20) | 174.17 (107.77) | 0.038 | 179.23 (109.82) | 178.40 (110.10) | 0.008 |
| **Before Imputation^c^** | | | | | | |
| HbA1c, %, mean(sd) | 8.07 (1.93) | 8.28 (1.78) | 0.113 | - | - | - |
| BMI, kg/m^2^, mean(sd) | 35.40 (6.20) | 31.41 (6.30) | 0.638 | - | - | - |
| Systolic blood pressure, mmHg, mean(sd) | 130.82 (15.56) | 131.63 (17.43) | 0.049 | - | - | - |
| Diastolic blood pressure, mmHg, mean(sd) | 77.40 (9.88) | 74.69 (10.77) | 0.262 | - | - | - |
| eGFR, mL/min/1.73m², mean(sd) | 81.85 (26.82) | 71.57 (29.86) | 0.362 | - | - | - |
| High-density lipoprotein cholesterol, mg/dL, mean(sd) | 41.57 (15.22) | 40.57 (15.86) | 0.064 | - | - | - |
| Low-density lipoprotein cholesterol, mg/dL, mean(sd) | 94.91 (36.13) | 90.46 (35.65) | 0.124 | - | - | - |
| Total cholesterol, mg/dL, mean(sd) | 173.52 (43.85) | 167.83 (44.11) | 0.129 | - | - | - |
| Triglycerides, mg/dL, mean(sd) | 180.94 (110.24) | 177.58 (109.42) | 0.031 | - | - | - |

^a^ Values are numbers (percentages) unless otherwise indicated.

^b^ Missing data were imputed using multiple imputation by chained equations, including all baseline covariates

^c^ The proportion of missing values for HbA1c, BMI, systolic blood pressure, diastolic blood pressure, eGFR, high-density lipoprotein cholesterol, low-density lipoprotein cholesterol, total cholesterol, triglycerides were 41.1%, 52.7%, 34.6%, 34.6%, 50.4%, 61.6%, 64.8%,62.6%, and 63.2%.

Abbreviations: GLP-1RA, glucagon-like peptide-1 receptor agonist; SGLT2is, sodium–glucose cotransporter-2 inhibitors; DPP4is, dipeptidyl peptidase-4 inhibitor; SMD, standardized mean difference; ACEi, angiotensin-converting enzyme inhibitor; BMI, body mass index; NSAIDs, nonsteroidal anti-inflammatory drugs; HIV/AIDS, human immunodeficiency virus/acquired immunodeficiency syndrome; eGFR, estimated glomerular filtration rate

**Supplement Figure 1.** The flowchart of patient selection in TriNetX electronic health record data

GLP-1RA, glucagon-like peptide-1 receptor agonists; SGLT2i, sodium-glucose cotransporter 2 inhibitors; PSM, propensity score matching

Patients were excluded (n= 1,070,384):

- No birth dates (n= 54,631)
- No using GLP-1RA, SGLT2i, or DPP4i (n=620,219)
- Multiple drug classes at entry (n=151,080)
- No encounter before index date (n=206,050)
- Age < 18 years (n=2,961)
- Outside study period (n=35,443)

Patients with type 2 diabetes in TriNetX up to Jan 31, 2025 (n= 2,124,534)

GLP-1RA, SGLT2i, or DPP4i users between Jan 1, 2014 and Jan 31, 2025 (n= 1,054,150)

Patients were excluded (n=40,641):

- Prior diagnosis of type 1 diabetes (n=16,004)
- Prior diagnosis of end-stage kidney disease/dialysis (n=8,531)
- Prior diagnosis of melanoma or nmsc (n=5,392)
- Incorrected documented date (e.g, prescription date>last encounter date or death date) (n=10,174)

GLP-1RA or SGLT2i users (n=761,917)

GLP-1RA or DPP4i users (n=682,392)

Prior user (n=37,720)

GLP-1RA or SGLT2i initiators (n=724,197)

●GLP-1RA (n=407,709)

●SGLT2i (n=316,488)

1:1 PSM:

●GLP-1RA (n=235,797)

●SGLT2i (n=235,797)

Prior user (n=34,298)

GLP-1RA or DPP4i initiators (n=648,094)

●GLP-1RA (n=408,393)

●DPP4i (n=239,701)

1:1 PSM:

●GLP-1RA (n=153,873)

●DPP4i (n=153,873)

GLP-1RA, SGLT2i, or DPP4i users (n=1,013,509)

**Supplement Figure 2.** The propensity score distribution before and after propensity score matching. DPP4i, dipeptidyl peptidase 4 inhibitors; GLP-1RA, glucagon-like peptide-1 receptor agonists; SGLT2i, sodium-glucose cotransporter 2 inhibitors.
